# Guiding Classical Biological Control of an Invasive Mealybug Using Integrative Taxonomy

**DOI:** 10.1371/journal.pone.0128685

**Published:** 2015-06-05

**Authors:** Aleixandre Beltrà, Pia Addison, Juan Antonio Ávalos, Didier Crochard, Ferran Garcia-Marí, Emilio Guerrieri, Jan H. Giliomee, Thibaut Malausa, Cristina Navarro-Campos, Ferran Palero, Antonia Soto

**Affiliations:** 1 Institut Agroforestal Mediterrani, Universitat Politècnica de València, València, Spain; 2 Department of Conservation Ecology and Entomology, Stellenbosch University, Stellenbosch, South Africa; 3 INRA, Univ. Nice Sophia Antipolis, CNRS, UMR 1355–7254 Institut Sophia Agrobiotech, 06900 Sophia Antipolis, France; 4 Istituto per la Protezione Sostenibile delle Piante, Consiglio Nazionale delle Ricerche, Portici, Italy; 5 Centre for Invasion Biology, Department of Botany & Zoology, Stellenbosch University, Stellenbosch, South Africa; University of Pretoria, SOUTH AFRICA

## Abstract

*Delottococcus aberiae* De Lotto (Hemiptera: Pseudococcidae) is a mealybug of Southern African origin that has recently been introduced into Eastern Spain. It causes severe distortions on young citrus fruits and represents a growing threat to Mediterranean citrus production. So far, biological control has proven unsatisfactory due to the absence of efficient natural enemies in Spain. Hence, the management of this pest currently relies only on chemical control. The introduction of natural enemies of *D*. *aberiae* from the native area of the pest represents a sustainable and economically viable alternative to reduce the risks linked to pesticide applications. Since biological control of mealybugs has been traditionally challenged by taxonomic misidentification, an intensive survey of *Delottococcus* spp. and their associated parasitoids in South Africa was required as a first step towards a classical biological control programme. Combining morphological and molecular characterization (integrative taxonomy) a total of nine mealybug species were identified in this study, including three species of *Delottococcus*. Different populations of *D*. *aberiae* were found on wild olive trees, in citrus orchards and on plants of *Chrysanthemoides monilifera*, showing intra-specific divergences according to their host plants. Interestingly, the invasive mealybug populations from Spanish orchards clustered together with the population on citrus from Limpopo Province (South Africa), sharing COI haplotypes. This result pointed to an optimum location to collect natural enemies against the invasive mealybug. A total of 14 parasitoid species were recovered from *Delottococcus* spp. and identified to genus and species level, by integrating morphological and molecular data. A parasitoid belonging to the genus *Anagyrus*, collected from *D*. *aberiae* in citrus orchards in Limpopo, is proposed here as a good biological control agent to be introduced into Spain.

## Introduction

Many mealybug species (Hemiptera: Pseudococcidae) are major pests that cause significant losses in crops and ornamental plants [1]. Due to their small size and cryptic behaviour, they are often unnoticed and become invasive species that spread through the international trade of fruits and ornamentals [2,3]. Population outbreaks are frequent when mealybugs are introduced into new areas without their specific natural enemies and therefore classical biological control programmes have been widely used for their management [4]. These programmes generally rely on the importation of encyrtid parasitoids from their native area, given that their high specificity enables optimum results to be achieved with low risk of parasitizing non-target hosts [4–6]. Encyrtid parasitoids have allowed for the effective control of important mealybug outbreaks, such as *Maconellicoccus hirsutus* (Green) [7] and *Paracoccus marginatus* Williams & Granara de Willink in the Caribbean [8,9] and *Rastrococcus invadens* Williams in West Africa [10,11]. Besides these successes, there are well-documented examples of biological control programmes against mealybugs that have failed [4,5,12]. According to Moore [4], the most common causes of these failures are host misidentification, hyperparasitism and low acclimation capacity. Taxonomy has proven to be crucial in mealybug biological control and the difficulties associated with morphological identification have delayed the implementation of several control programmes. For example, the misidentification of *Planococcus kenyae* (Le Pelley) as *P*. *lilacinus* (Cockerell) led to the unsuccessful introduction of several parasitoids from Southeast Asia into Kenya [5]. Similarly, the misidentification of *Phenacoccus manihoti* Matile-Ferrero led to the ineffective introduction of *Phenacoccus herreni* Cox & Williams parasitoids into West Africa [13]. Both cases were later amended through the correct identification of the target mealybugs and the introduction of host-specific parasitoids [5,14]. Taxonomic expertise is also required to identify the candidate species of parasitoids for biological control [15]. Indeed, some biological and behavioural characteristics relevant for biological control such as host preference may differ in closely-related natural enemies [16].

Morphological identification of mealybugs and encyrtids share similar difficulties: high number of undescribed species, reduced number of experienced taxonomists and presence of cryptic species. These difficulties can be addressed by applying integrative taxonomy, which combines multiple disciplines such as phylogeography, comparative anatomy, population genetics, ecology and behavioural biology to solve taxonomic problems and delimit species [17,18]. In recent years, the integration of molecular techniques for the characterization of mealybugs and encyrtids has provided a new approach for correct identification at species level [19–25]. Among these techniques, DNA barcoding has been shown to be particularly useful because it allows for fast and accurate identification of previously sequenced species, in addition to the flagging of cryptic species and providing important insights into population genetics and molecular phylogenetics [26,27]. From an applied point of view, DNA barcoding can be a key tool for assessing the specific area of origin of the target pest and selecting its coevolved natural enemies [28–30].

*Delottococcus aberiae* (De Lotto) (Hemiptera: Pseudococcidae) is an invasive mealybug from Southern Africa that was detected in Eastern Spain in 2009 causing serious damage in citrus orchards [31]. This polyphagous species feeds on tropical and subtropical crops such as coffee, guava, citrus, persimmon, and pear [32–34]. Like other species of mealybugs damaging citrus, *D*. *aberiae* reduces plant vigour and excretes honeydew which promotes the growth of sooty-mould fungi. However, when *D*. *aberiae* develops on young fruits it causes severe distortions leading to major crop losses. Since its establishment, surveys in citrus orchards revealed the absence of parasitoids and the inadequacy of generalist predators for controlling outbreaks of *D*. *aberiae* in spring and summer [35]. Thus, the management of *D*. *aberiae* still relies on the application of broad-spectrum insecticides such as chlorpyrifos. The economic and environmental impacts of chemical control, and its potential interference with the biological control of other citrus pests, compelled us to develop additional management strategies. Among them, classical biological control, which has been successfully used against invasive scale insects in Spanish citrus [36,37], appeared feasible and affordable.

The success of an effective biological control program for *D*. *aberiae* could be challenged by the misidentification of the mealybug and/or its natural enemies. Indeed, Miller and Giliomee [34] suggested the presence of cryptic species within morphospecies of *D*. *aberiae* and only one parasitoid has been tentatively associated with *D*. *aberiae* [38]. Therefore, this study was a first step towards classical biological control of *D*. *aberiae*, in which we surveyed mealybug populations in Spanish and South African citrus orchards and natural ecosystems to characterize *Delottococcus* spp. and their parasitoids. Specifically, we used integrative taxonomy to: i) discriminate *D*. *aberiae* and closely related species; ii) estimate the intraspecific genetic distances among populations of *D*. *aberiae*; and iii) identify candidate parasitoids for biological control of *Delottococcus* species.

## Materials and Methods

### Mealybug and parasitoid survey

A total of 25 sites were surveyed across Eastern Spain and the South African provinces of the Western Cape, Mpumalanga and Limpopo, between 2012 and 2014 (Table 1). Sampling sites comprised natural ecosystems, citrus orchards and botanical gardens. Some of these sites were selected following previous records of *Delottococcus* spp. [34]. Mealybugs were collected and placed in small plastic vials with 70% ethanol and preserved at -20°C for molecular identification. When populations of *Delottococcus* were tentatively identified in the field, mealybug infested twigs and leaves were collected for two hours. The material was placed into sampling bags and examined in the laboratory with a dissecting microscope. Mummified mealybugs were isolated to 3 x 0.8 cm glass vials covered with a cotton plug and kept in the laboratory at room temperature (20 ± 5°C) and natural photoperiod. Vials were checked daily for parasitoid emergence. Upon emergence, 70% ethanol was added into the tube to kill adult parasitoids and vials were stored at -20°C.

**Table 1:** Collection localities, mealybugs and parasitoids surveyed from South Africa and Spain.

Sampling site						Mealybugs		Parasitoids
Province	City	Host plant	GPS coordinates	Protection status	Collection date	Population	Species	Species
Western Cape	Stellenbosch	*Olea europaea subsp*. *africana*	-33.945104,18.842711	Non protected area	25/01/2012	1	*Delottococcus aberiae*	*Anagyrus aurantifrons* (2)
	Stellenbosch	*Olea europaea subsp*. *africana*	-33.942719,18.859448	Non protected area	28/01/2012	2	*Delottococcus aberiae*	*Lamennaisia* sp. (5)
	Stellenbosch	*Olea europaea subsp*. *africana*	-33.933266,18.886614	Non protected area	2/02/2012	3	*Delottococcus aberiae*	*Pachyneuron* sp. (1)
	Stellenbosch	*Olea europaea subsp*. *africana*	-33.940886, 18.858011	Non protected area	8/02/2012	4	*Delottococcus aberiae* (6)	*Aenasius comperei* (3)
	Stellenbosch	*Olea europaea subsp*. *africana*	-33.93834,18.879361	Non protected area	9/02/2012	5	*Delottococcus aberiae*	*Anagyrus aurantifrons* (2), Cynipoidea (1), *Lamennaisia sp*. (1), Proctotrupoidea (2)
	Stellenbosch	*Olea europaea subsp*. *africana*	-33.93729,18.875188	Non protected area	26/02/2012	6	*Delottococcus aberiae*	*Anagyrus aurantifrons* (5)
	Stellenbosch	*Olea europaea subsp*. *africana*	-33.929616, 18.851437	Non protected area	12/03/2012	7	*Delottococcus aberiae* (6)	-
	Kirstenbosch	*Olea europaea subsp*. *africana*	-33.986828,18.435936	Non protected area	3/02/2012	8	*Delottococcus aberiae*	-
	Kirstenbosch	*Olea europaea subsp*. *africana*	-33.986828,18.435936	Non protected area	28/02/2012	9	*Delottococcus aberiae*	-
	Jonkershoek	*Olea europaea subsp*. *africana*	-33.968122,18.933896	Nature reserve	31/01/2012	10	*Delottococcus aberiae*	-
	Paarl	*Olea europaea subsp*. *africana*	-33.762416,18.933198	Non protected area	16/02/2012	11	*Delottococcus aberiae*	-
	Wellington	*Olea europaea subsp*. *africana*	-33.7775, 18.951111	Non protected area	28/02/2012	12	*Delottococcus aberiae*	-
	Citrusdal	*Citrus sinensis*	-32.61393,18.709717	Private land	26/01/2012	13	*Planococcus citri*	-
	Citrusdal	*Citrus sinensis*	-32.41127,18.790741	Private land	26/01/2012	14	*Planococcus citri*	-
	Stellenbosch	*Citrus sinensis*	-33.944624,18.870885	Non protected area	12/02/2012	15	*Pseudococcus longispinus; Planococcus citri*	-
	Vermont	*Chrysanthemoides monilifera*	-34.415478,19.177537	Private land	1/01/2011	16	*Delottococcus aberiae* (7)	-
	Vermont	*Chrysanthemoides monilifera*	-34.415478,19.177537	Private land	28/02/2012	17	*Vryburgia transvaalensis* (4)	*Coccophagus* sp. (1)
	Jonkershoek	*Phylica pubescens*	-33.968122,18.933896	Nature reserve	5/02/2012	18	*Delottococcus phylicus* (6)	*Anagyrus sp*. 2 (1), *Chartocerus* sp. 1 (8), *Rhopus notuis* (4), *Anagyrus sp*. 1 (1)
	Kirstenbosch	*Phylica pubescens*	-33.982513,18.453941	Non protected area	28/02/2012	19	*Delottococcus phylicus*	*Anagyrus* sp. 1 (2), Proctotrupoidea (1)
	Kirstenbosch	*Leucadendron argenteum*	-33.982513,18.453941	Non protected area	28/02/2012	20	*Delottococcus confusus* (6)	*Dendrocerus* sp. (1), *Prochyloneurus sp*. (1)
	Porterville	*Protea magnifica*	-32.931433,19.040717	Private land	6/03/2012	21	*Delottococcus confusus* (6)	*Chartocerus* sp. 1 (1), *Chartocerus sp*. 2 (1)
Mpumalanga	Nelspruit	*Citrus sinensis*	-25.4759,31.003375	Private land	1/03/2012	22	*Paracoccus burnerae* (6)	-
	Nelspruit	*Citrus spp*.	-25.435485,30.970631	Private land	1/03/2012	23	*Paracoccus burnerae*	-
	Nelspruit	*Citrus spp*.	-25.435485,30.970631	Private land	2/03/2012	24	*Ferrisia virgata*	-
	Nelspruit	*Citrus spp*.	-25.435485,30.970631	Private land	2/03/2012	25	*Planococcus citri*	-
	Nelspruit	*Citrus spp*.	-25.435485,30.970631	Private land	2/03/2012	26	*Paracoccus burnerae*	-
	Nelspruit	*Olea europaea*	-25.462495,30.94677	Non protected area	2/03/2012	27	*Nairobia bifrons*	-
Limpopo	Letsitele	*Citrus x paradisi*	-23.853205,30.388875	Private land	29/01/2014	28	*Delottococcus aberiae* (2)	-
	Letsitele	*Citrus x paradisi*	-23.848194,30.401205	Private land	29/01/2014	29	*Delottococcus aberiae*	-
	Letsitele	*Citrus x paradisi*	-23.798969,30.436491	Private land	29/01/2014	30	*Delottococcus aberiae* (3)	*Anagyrus* sp. 1 (3)
	Letsitele	*Citrus x paradisi*	-23.839636,30.453972	Private land	29/01/2014	31	*Delottococcus aberiae* (3)	-
Comunitat Valenciana	Quart de les Valls	*Citrus reticulata x Citrus sinensis*	39.745544, -0.296638	Private land	16/07/2012	32	*Delottococcus aberiae* (5)	-

Number of individuals sequenced (n).

All samplings were carried out on private land and non-protected areas, except those conducted in Jonkershoek Nature Reserve which were under permit number 0056-AAA041 00028 from Cape-Nature (Table 1). All private properties were surveyed under permission of their owners. No specific permission was required for sampling insects in other areas. The samplings did not involve endangered or protected species.

### Morphological and molecular characterization of insects

The characterization of mealybugs and parasitoids was carried out in the following steps: i) morphological identification of all the mealybug populations surveyed examining five individuals of each population; ii) molecular analysis of *Delottococcus* and closely-related genera; iii) molecular analysis of parasitoid specimens emerged from *Delottococcus* populations; iv) morphological identification of all the mealybug and parasitoid specimens whose DNA was successfully sequenced.

Mealybug morphological identification was performed according to the procedures described by Williams & Granara de Willink [39] with modifications. A small ventral incision was cut behind the hind leg of each specimen and it was heated to 80°C in KOH for 25 minutes and washed in distilled water for 15 minutes. Once the body contents were removed, the specimen was stained for one hour in a saturated solution of fuchsin in a 1:1:1 mixture of water, lactic acid and glycerol. Following this, the specimen was transferred into acetic acid for one hour to fix the dye and then moved into clove oil for one hour. Insects were slide-mounted in Canada balsam. Mealybugs were identified to species level using available taxonomic keys [32,34,40–43]. The slides are available for examination at the Polytechnic University of Valencia (Valencia, Spain) for any interested researcher who visited this location.

Parasitoid identification was carried out as follows: Every specimen sequenced was placed for 24h in a xylene and absolute ethanol 1:1 solution, transferred into amyl acetate for 24 h, dried up in amyl acetate until evaporation and mounted on card with water-soluble glue. Selected card-mounted specimens were slide-mounted following Noyes [44]. In brief, wings were removed from card-mounted specimens and mounted in Canada balsam with no further passage. The remaining insect was transferred for 10 minutes to a KOH 10% solution at 100°C, moved to acetic acid for five minutes at room temperature, rinsed with distilled water and dehydrated in a progressive series of ethanol (from 70% to 100%). Once in absolute ethanol a drop of clove oil was added waiting the complete evaporation of ethanol. The insect was then transferred to the slide in a Canada balsam drop, dissected and heated at 100° overnight. All slides are deposited at Università degli Studi di Napoli Federico II (Portici, Italy) and are available for examination for any interested researcher who visited this location. Parasitoids were identified to species or genus level with the aid of available keys and by comparing them with type material or material authoritatively identified and preserved at the Natural History Museum of London (UK).

Ten mealybug populations and 44 parasitoid specimens were selected for further molecular analyses (Table 1). DNA was extracted without crushing the specimen body using the DNeasy Tissue Kit (QIAGEN, Hilden, Germany) [20] for mealybugs and the prepGEM Insect Kit (ZyGEM, Lane Hamilton, New Zealand) for parasitoids. DNA was amplified from three different genes: the cytochrome oxidase subunit 1 (mtDNA), the 28S ribosomal gene (nuclear genome), and the 16s-leuA region (from the genome of the mealybug endosymbiont *Tremblaya princeps*) (Table 2). PCR was performed with a 23μl reaction mixture and 2μl of diluted DNA (1–20 ng). The reagent concentrations were 12.5μl of 1X QIAGEN Multiplex PCR buffer and 0.2μM of each primer (primers in Table 2). PCR was carried out as follows: initial denaturation at 95°C for 15 minutes, followed by 35 cycles of denaturation at 94°C for 30s, annealing for 90s at a temperature of 50°C–60°C, depending on the primers (Table 2), elongation at 72°C for 60s, followed by a final extension at 72°C for 10 minutes. The final products were separated by electrophoresis with the QIAxcel Advanced System (QIAGEN, Hilden, Germany) for quality checking. PCR products were then sequenced in both directions using an ABI 3130XL automatic sequencer (Applied Biosystems, Foster City, CA, USA) at Genoscreen (Lille, France) or Beckman Genomics (Takeley, United Kingdom). Consensus sequences were generated and analysed with Seqscape v2.5 software (Applied Biosystems), and alignments were manually edited with Bioedit [45]. When a sequence of a specimen displayed genetic variation at one or more site(s), it was considered as a new haplotype. The analysed sequences were deposited in Genbank under accession numbers as shown in S1 and S2 Tables.

**Table 2:** PCR primers used in this study to amplify mealybug and parasitoid DNA.

Target group	Locus	Primer names	Primer sequences	Annealing temperature	PCR product length (bp)	Reference
**Pseudococcidae**	COI	PcoF1-LepR1	CCTTCAACTAATCATAAAAATATYAG / TAAACTTCTGGATGTCCAAAAAATCA	54°C	~700bp	[61,62]
	28S (D10)	S3690-A4394	GAGAGTTMAASAGTACGTGAAAC / TCGGARGGAACCAGCTACTA	58°C	~800bp	[63]
	rpS15-16ST	leuA-U16S	GTATCTAGAGGNATHCAYCARGAYGGNG / GCCGTMCGACTWGCATGTG	60°C	~1000bp	[64]
**Chalcidoidea**	COI	LCO1490-HCO2198	GGTCAACAAATCATAAAGATATTGG / TAAACTTCAGGGTGACCAAAAAATCA	50°C	~700bp	[65]
	28S (D2)	28S-D2 (F)- 28S-D2 (R)	CGTGTTGCTTGATAGTGCAGC / TCAAGACGGGTCCTGAAAGT	58°C	~600bp	[66]

### Mealybug phylogenetic analysis and intraspecific variability

In order to carry out the phylogenetic analysis, DNA sequences from representative species of other Pseudococcidae were obtained in our laboratory by sequencing or from public databases (S1 Table). Alignments of the sequence data-sets were conducted using the program Muscle v3.6 (Edgar, 2004) with default parameters. To avoid alignment ambiguity, gaps and hyper-variable regions were excluded using GBlocks [46] with the following parameters: minimum number of sequences for a conserved or flanking position: 32, maximum number of contiguous non-conserved positions: 8, minimum length of a block: 10, and allowed gap positions: with half. Single-gene alignments were then concatenated and the best-fit model of DNA evolution was selected in MEGA6 [47]. Models with the lowest BIC (Bayesian Information Criterion) scores are considered to better describe the DNA substitution pattern of our alignment. Non-uniformity of evolutionary rates among sites was modelled by using a discrete Gamma distribution (+G) with 5 rate categories and by assuming that a certain fraction of sites are evolutionarily invariable (+I). After selecting for the best-fit DNA substitution model, Bayesian inference was applied using the BEAST software [48] to infer phylogenetic relationships among samples. Two independent runs starting from a random tree, using estimated base frequencies under the best-fit model and a Yule tree prior were used. Markov chains were run for 10,000,000 generations, sampling every 1,000th tree. All MCMC runs were assessed using Tracer v1.5, the graphical tool for visualization and diagnostics of MCMC output, and with a 10% burn-in. Finally, the sample of trees obtained from the MCMC runs after discarding the burn-in was summarized onto a single consensus tree using TreeAnnotator [48].

The cytochrome oxidase subunit 1 (COI) is commonly used in DNA barcoding studies to distinguish between species and it has been shown to be particularly useful for mealybugs [20,21,49]. Therefore, the COI-gene intraspecific divergence among mealybug populations collected on different hosts was also estimated through Maximum Composite Likelihood [50]. Estimates of intraspecific divergence and the corresponding standard errors were obtained using MEGA6 [47].

## Results

### Mealybug species identified

A total of nine different mealybug species were identified from the 24 sites surveyed in South Africa (Table 1). Five mealybug species were collected from citrus orchards: *D*. *aberiae*, *Planococcus citri* (Risso), *Pseudococcus longispinus* (Targioni Tozzetti), *Paracoccus burnerae* (Brain) and *Ferrisia virgata* (Cockerell). Two species were recovered on olive trees: *D*. *aberiae* and *Nairobia bifrons* De Lotto, even though these species did not co-occur in the same location. *Delottococcus aberiae* was present on wild olive trees (*Olea europaea* subsp. a*fricana*) in Western Cape natural ecosystems, while *N*. *bifrons* was occasionally found on *Olea europaea* in Nelspruit botanical gardens. Moreover, other mealybug species were collected from different host plants: *Vryburgia transvaalensis* (Brain) and *D*. *aberiae* on *Chrysanthemoides monilifera*; *Delottococcus phylicus* (De Lotto) on *Phylica pubescens*; and *Delottococcus confusus* (De Lotto) on *Leucadendron argenteum* and *Protea magnifica*. New DNA sequences are provided for the species *D*. *aberiae*, *D*. *confusus*, *D*. *phylicus*, *P*. *burnerae* and *V*. *transvaalensis*.

### Mealybug phylogenetic analysis and intraspecific variability

**Fig 1:**
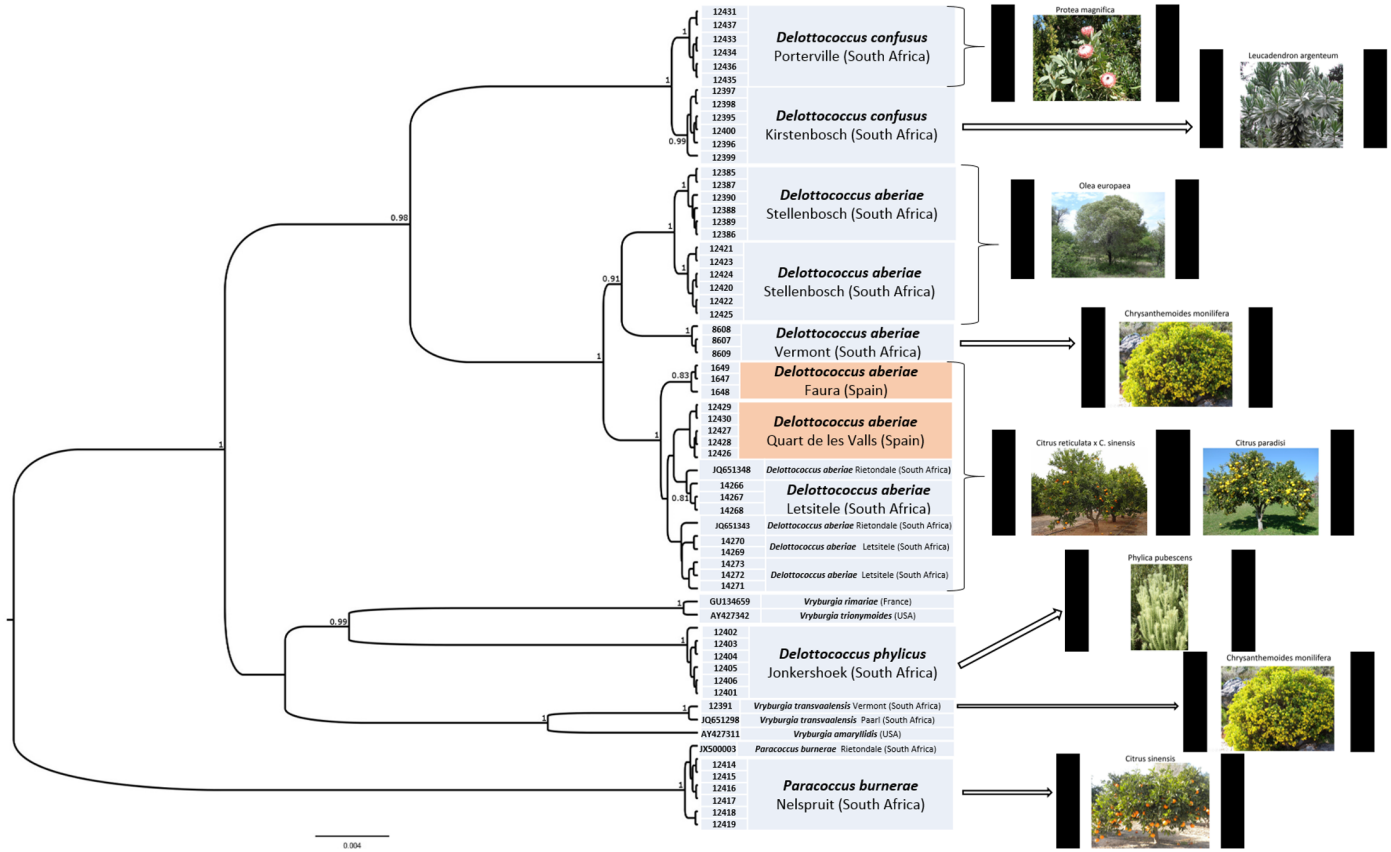
Phylogenetic relationships among the mealybug taxa surveyed in study, as revealed by a Bayesian consensus tree obtained using the BEAST package. For each sample of the dataset, the morphological identification, the host plant, the sampling site and the specimen code are given.

The dataset for the concatenated 28S, 16S and cytochrome oxidase alignments had an initial length of 2531 bp. A total of 2353 positions were kept for further analyses after running GBlocks (92% of the original 2531 positions). The nucleotide substitution model selected in MEGA6 was TrN93+G (BIC = 11720), with the estimated alpha parameter for the gamma distribution (α = 0.058) indicating a significant heterogeneity on the DNA substitution among sites. The effective sample size for each parameter under Bayesian inference was always >200, indicating a convergence of the MCMC runs. The consensus phylogenetic tree showed a highly significant clustering of all specimens of *D*. *aberiae*, with a significant support for the monophyly of this clade (Fig 1). Reciprocal monophyly was found among populations of *D*. *aberiae* obtained from different host plants. The Spanish populations of *D*. *aberiae* collected from citrus clustered significantly with the South African populations from citrus in the Limpopo Province. The specimens of *D*. *confusus* collected in our study also present strongly supported clades that correspond to mealybugs collected from different host plants. However, and contrary to the case of *D*. *aberiae*, host plant and geography cannot be disentangled within our dataset for *D*. *confusus*. Finally, *D*. *phylicus* was found to cluster with species belonging to the genus *Vryburgia*.

The Maximum Composite Likelihood genetic divergence among different species of *Delottococcus* ranged between 5.3% and 6.4% (Table 3). Genetic divergence was estimated among populations of *D*. *aberiae* to further characterize intraspecific patterns according to plant hosts. Specifically, citrus populations diverged 1.1% from those on wild olive trees and 1.8% from those on *C*. *monilifera*. These values are much larger than those found among populations of *D*. *confusus* from *L*. *argenteum* and *P*. *magnifica* (divergence = 0.2%).

**Table 3:** Estimates of evolutionary divergence over sequence pairs between groups.

Num.	Mealybug	Host	Num.							
			1	2	3	4	5	6	7	8
1	*Delottococcus aberiae*	*Olea europaea*		**0.003**	**0.003**	0.005	0.006	0.009	0.014	0.010
2	*Delottococcus aberiae*	*Citrus* x *paradisi*	**1.1%**		**0.005**	0.009	0.009	0.012	0.014	0.012
3	*Delottococcus aberiae*	*Chrysanthemoides monilifera*	**1.2%**	**1.8%**		0.009	0.009	0.011	0.015	0.012
4	*Delottococcus confusus*	*Protea magnifica*	3.0%	4.8%	4.9%		0.001	0.009	0.013	0.011
5	*Delottococcus confusus*	*Leucadendron argenteum*	3.2%	4.7%	4.6%	0.2%		0.009	0.013	0.011
6	*Delottococcus phylicus*	*Phylica pubescens*	5.3%	6.4%	6.2%	5.8%	5.7%		0.010	0.012
7	*Vryburgia transvaalensis*	*Chrysanthemoides monilifera*	5.9%	6.1%	6.4%	5.7%	5.8%	5.0%		0.017
8	*Paracoccus burnerae*	*Citrus sinensis*	7.3%	7.4%	7.6%	8.1%	7.7%	8.2%	8.5%	

Standard error estimate(s) are shown above the diagonal. Numbers in bold denote the estimates between populations of *Delottococcus aberiae*.

### Parasitoid species identified

Fourteen parasitoid species emerged from *Delottococcus* spp. (Table 1). Most of these parasitoids were recovered from populations of *D*. *aberiae*, namely: four species of Encyrtidae, *Anagyrus aurantifrons* Compere (new host record), *Anagyrus* sp. 1 (new host record), *Aenasius comperei* (Kerrich), *Lamennaisia* sp. (new host record); one species of Pteromalidae, *Pachyneuron* sp.; and two species of the superfamily Proctotrupoidea and the family Cynipidae. Two species of *Anagyrus* (*Anagyrus* sp. 1 and *Anagyrus* sp. 2) were found parasitizing *D*. *phylicus* together with *Rhopus notius* Prinsloo (Encyrtidae) (new host records), *Chartocerus* sp. (Signiphoridae) and one species of Proctotrupoidea. Finally, *Prochiloneurus* sp. (Encyrtidae) (new host record), two species of *Chartocerus* (Signiphoridae) and *Dendrocerus* sp. (Ceraphronoidea) were collected from *D*. *confusus* (Table 1). All these species were processed with molecular analysis and we obtained 59 sequences characterizing these specimens (accession numbers in S2 Table).

## Discussion

The success of a biocontrol programme against *D*. *aberiae* in Spanish citrus orchards could be impaired by the misidentification of either mealybug and/or its natural enemies. The main aim of the current research was to characterize the diversity of *D*. *aberiae* and closely-related species with that of their natural enemies, collected from the native area of the mealybug. A total of nine mealybug species were identified in this survey, three of them belonging to the genus *Delottococcus* (i.e. *D*. *aberiae*, *D*. *phylicus* and *D*. *confusus*). In the Western Cape area (SW within South Africa), *D*. *aberiae* was mainly found on wild olive trees (homogeneously distributed at low densities) and on the roots of the flowering shrub *Chrysanthemoides monilifera* as reported by Miller and Giliomee [34], but it could not be found in citrus orchards. However, populations of *D*. *aberiae* were successfully recovered from citrus orchards (*Citrus x paradisi*) in the Limpopo area (NE within South Africa) where some outbreaks had been previously detected by Citrus Research International (Table 1). The irregular distribution of *D*. *aberiae* in South African citrus orchards was expected, considering that in this country it is a secondary pest of citrus that can go unnoticed for years [33,34].

Molecular data on *Delottococcus* and *Vryburgia* are scarce, so our results represent an important contribution to characterize the diversity of South African mealybugs. Hardy et al. [51] proposed for the first time the existence of a South African clade composed by the genera *Diversicrus* De Lotto, *Vryburgia* De Lotto, *Lenania* De Lotto, and some species of the paraphyletic genera *Paracoccus* Ezzat & McConnell, *Paraputo* Laing, and *Erium* Cockerell. In a preliminary study, Beltrà et al. [31] also found that the introduced populations of *D*. *aberiae* from Spain were closely related to those of introduced *Vryburgia rimariae* Tranfaglia from France, which reinforced the idea of a South African clade. The existence of this clade is supported by our study, following an intensive survey of South African mealybugs, which included populations of several species of *Delottococcus*. The Bayesian phylogenetic tree confirmed that *Delottococcus* and *Vryburgia* are paraphyletic genera, which is in agreement with the fact that none of the characters used to define *Delottococcus* are consistently present in all the species of this genus [34,52]. These sequences will enable inexperienced taxonomists to perform precise identifications using DNA comparison and therefore contribute to the characterization of some South African mealybugs as a complement to the initial works of Pieterse et al. [53] and Sethusa et al. [54]. The new sequences could also be used in further studies to develop a multiplex PCR protocol for fast identification of citrus mealybugs in quarantine controls including the South African species *D*. *aberiae* and *P*. *burnerae*.

The intraspecific variation found among populations of *D*. *aberiae* ranged from 1.1% to 1.8% in the COI locus, which might not be high enough to state conclusively that the populations collected on citrus, wild olives and *C*. *monilifera* constitute different species. Although Park et al. [21] found an average intraspecific genetic divergence of 0.97% within scale insect species, one quarter of their species showed divergences larger than 2.0%. In another study, Rung et al. [49] reported intraspecific genetic divergences from 1.90% to 1.98% among cryptic species of genus *Planococcus*. Nevertheless, the variation found among populations of *D*. *aberiae* from South Africa should not be ignored, because it is a key aspect for the collection of specific and efficient natural enemies. Parasitoids are usually adapted to local host populations and can be more effective in parasitizing local genotypes [55]. Many encyrtids show specific interactions with mealybugs and their coevolution plays an important role on their ability to overcome defensive strategies of their hosts [6,56–58]. Indeed, specific strains of the encyrtids *Anagyrus* sp. near *pseudococci* (Girault) have shown to be more effective in parasitizing specific populations of *Planococcus ficus* (Signoret) [59]. Our results showed that populations of *D*. *aberiae* from Spain were closest to those found in Limpopo citrus orchards, sharing identical COI haplotypes. Therefore, within the framework of a classical biological control programme, this geographic area should be considered as a first choice for collecting parasitoids to be introduced into Europe against *D*. *aberiae*. Furthermore, the genetic analyses also provided some insights into the possible introduction pathway of *D*. *aberiae*, suggesting that fruit trade could have been involved in the mealybug invasion. This is in agreement with historical records showing that citrus fruit importation is one of the most frequent pathways of introduction of scale insects into Europe and that the first record of *D*. *aberiae* in Spain was located close to a citrus warehouse [3,35].

Parasitoid identification in the current study greatly benefited from combining molecular and morphological data analysis. This technique was particularly useful for matching males and females of different, though closely related, parasitoid species emerging from the same host. The parasitoid complex of *Delottococcus* spp. collected in this survey consisted of 14 parasitoid species. One species, namely *A*. *comperei*, has already been reported from *Delottococcus* spp. [38,60] whilst *Anagyrus*, *Lamennaisia*, *Rhopus*, and *Prochiloneurus* spp, are new records for *Delottococcus* spp. Our data integrate previous parasitoid records already available for *Delottococcus* spp. (Table 4). Among the parasitoids recovered, the species of *Anagyrus*, *Aenasius*, and *Rhopus* might be of special interest because these genera have been widely used in mealybug biological control. *Anagyrus* sp. 1 should be considered as the most promising biological control candidate for introduction into Eastern Spain because it parasitized the haplotypes of *D*. *aberiae* found in South African citrus orchards. Further research includes the detailed taxonomical characterization of this species and the completion of laboratory bioassays to assess its host specificity and performance on parasitizing Spanish haplotypes of *D*. *aberiae*.

**Table 4:** Parasitoids of *Delottococcus* spp. recorded in previous works.

Mealybug host	Parasitoid species
*Delottococcus sp*.	*Aenasius comperei* [13]
*Delottococcus sp*.	*Gyranusoidea klugei* [67]
*Delottococcus sp*.	*Gyranusoidea litura*[68,69]
*Delottococcus sp*.	*Pseudococcobius dolus* [70]
*Delottococcus sp*.	*Pseudococcobius vibex* [70]
*Delottococcus sp*.	*Aenasius sp*. [69]
*Delottococcus sp*.	*Anagyrus sp*. [69]
*Delottococcus sp*.	*Anagyrus nigrescens* [71]
*Delottococcus sp*.	*Aphycus sp*. [69]
*Delottococcus aberiae*	*Aenasius comperei* [13]
*Delottococcus proteae*	*Leptomastix dactylopii* [72]
*Delottococcus quaesitus*	*Aenasius comperei* [13]
*Delottococcus quaesitus*	*Cheiloneurus carinatus* [69]
*Delottococcus quaesitus*	*Coccidoxenoides perminutus* [69]
*Delottococcus quaesitus*	*Gyranusoidea citrina* [68,69]
*Delottococcus quaesitus*	*Leptomastidea usta* [73]
*Delottococcus quaesitus*	*Leptomastix dactylopii* [72,74]
*Delottococcus quaesitus*	*Aphycus sp*. [69]
*Delottococcus quaesitus*	*Anagyrus sp*. [69]
*Delottococcus trichiliae*	*Aenasius sp*. [69]
*Delottococcus trichiliae*	*Anagyrus sp*. [69]
*Delottococcus trichiliae*	*Clauselina sp*. [69]
*Delottococcus taigae*	*Leptomastidea bifasciata* [75]

## Supporting Information

S1 Table Complete list of mealybug samples with corresponding haplotypes: codes of voucher slide mounted specimens, species, population code (see Table 1) and Genbank accession numbers for haplotypes. (DOCX)

S2 Table Complete list of parasitoid samples with corresponding haplotypes: codes of voucher slide-mounted specimens, species, population code (see Table 1) and Genbank accession numbers for haplotypes. (DOCX)
