# Supplementary material for: Guiding Classical Biological Control of an Invasive Mealybug Using Integrative Taxonomy
Source: PLoS One. 2015 Jun 5;10(6):e0128685. doi: 10.1371/journal.pone.0128685 (PMC4457817; doi:10.1371/journal.pone.0128685)
Supplement: S1 Table — (DOCX) [file pone.0128685.s001.docx]

| **Specimen Id.** | **Species** | **Population** | **Genbank Accession numbers** | | |
| --- | --- | --- | --- | --- | --- |
|  |  |  | **28S** | **LCO** | **16S** |
| 8607 | *Delottococcus aberiae* | 16 | KP771933 | KP771951 | - |
| 8608 |  |  | KP771933 | KP771951 | - |
| 8609 |  |  | KP771933 | KP771951 | - |
| 12385 | *Delottococcus aberiae* | 7 | KP771926 | KP771950 | KP771967 |
| 12386 |  |  | KP771926 | KP771950 | KP771967 |
| 12387 |  |  | KP771926 | KP771950 | KP771967 |
| 12388 |  |  | KP771926 | KP771950 | KP771967 |
| 12389 |  |  | KP771926 | KP771950 | KP771967 |
| 12390 |  |  | KP771926 | KP771950 | KP771967 |
| 12391 | *Vryburgia transvaalensis* | 17 | KP771934 | - | - |
| 12392 |  |  | - | - | - |
| 12393 |  |  | - | - | - |
| 12394 |  |  | - | - | - |
| 12395 | *Delottococcus confusus* | 20 | KP771927 | KP771952 | KP771969 |
| 12396 |  |  | KP771927 | KP771952 | KP771969 |
| 12397 |  |  | KP771927 | KP771952 | KP771969 |
| 12398 |  |  | KP771927 | KP771952 | - |
| 12399 |  |  | KP771927 | KP771952 | - |
| 12400 |  |  | KP771927 | KP771952 | KP771969 |
| 12401 | *Delottococcus phylicus* | 18 | KP771928 | KP771954 | KP771971 |
| 12402 |  |  | KP771928 | KP771954 | - |
| 12403 |  |  | KP771928 | KP771954 | - |
| 12404 |  |  | KP771928 | KP771954 | - |
| 12405 |  |  | KP771928 | KP771954 | KP771971 |
| 12406 |  |  | KP771928 | KP771954 | KP771971 |
| 12414 | *Paracoccus burnerae* | 22 | KP771929 | KP771955 | KP771972 |
| 12415 |  |  | KP771929 | KP771955 | - |
| 12416 |  |  | KP771929 | KP771955 | KP771972 |
| 12417 |  |  | KP771929 | KP771955 | KP771972 |
| 12418 |  |  | KP771929 | KP771955 | - |
| 12419 |  |  | KP771929 | KP771955 | KP771972 |
| 12420 | *Delottococcus aberiae* | 4 | KP771926 | KP771950 | KP771968 |
| 12421 |  |  | KP771926 | KP771950 | KP771968 |
| 12422 |  |  | KP771926 | KP771950 | KP771968 |
| 12423 |  |  | KP771926 | KP771950 | KP771968 |
| 12424 |  |  | KP771926 | KP771950 | KP771968 |
| 12425 |  |  | KP771926 | KP771950 | KP771968 |
| 12426 | *Delottococcus aberiae* | 32 | KP771930 | KP771956 | KP771973 |
| 12427 |  |  | KP771930 | KP771956 | KP771973 |
| 12428 |  |  | KP771930 | KP771956 | KP771973 |
| 12429 |  |  | KP771930 | KP771956 | KP771973 |
| 12430 |  |  | KP771930 | KP771956 | KP771973 |
| 12431 | *Delottococcus confusus* | 21 | KP771931 | KP771953 | KP771970 |
| 12433 |  |  | KP771931 | KP771953 | KP771970 |
| 12434 |  |  | KP771931 | KP771953 | KP771970 |
| 12435 |  |  | KP771931 | KP771953 | KP771970 |
| 12436 |  |  | KP771931 | KP771953 | KP771970 |
| 12437 |  |  | KP771931 | KP771953 | KP771970 |
| 14266 | *Delottococcus aberiae* | 31 | KP771932 | KP771958 | - |
| 14267 |  |  | KP771932 | KP771958 | - |
| 14268 |  |  | KP771932 | KP771958 | - |
| 14269 | *Delottococcus aberiae* | 28 | KP771932 | KP771957 | - |
| 14270 |  |  | KP771932 | KP771957 | - |
| 14271 | *Delottococcus aberiae* | 30 | KP771932 | KP771957 | - |
| 14272 |  |  | KP771932 | KP771957 | - |
| 14273 |  |  | KP771932 | KP771957 | - |
| NCBI | *Delottococcus aberiae* | Pretoria, Gauteng | JQ651343 | - | - |
| NCBI | *Delottococcus aberiae* | Pretoria, Gauteng | JQ651348 | - | - |
| NCBI | *Paracoccus burnerae* | Pretoria, Gauteng | JX500003 | - | - |
| NCBI | *Vryburgia transvaalensis* | Paarl,  Western Cape | JQ651298 | - | - |
| NCBI | *Vryburgia rimariae* | France | GU134659 | GU134697 | - |
| NCBI | *Vryburgia trionymoides* | USA | AY427342 | - | - |
| NCBI | *Vryburgia amaryllidis* | USA | AY427311 | - | - |
