# Supplementary material for: Guiding Classical Biological Control of an Invasive Mealybug Using Integrative Taxonomy
Source: PLoS One. 2015 Jun 5;10(6):e0128685. doi: 10.1371/journal.pone.0128685 (PMC4457817; doi:10.1371/journal.pone.0128685)
Supplement: S2 Table — (DOCX) [file pone.0128685.s002.docx]

| **Specimen Id.** | **Species** | **Population** | **Genbank Accession** | |
| --- | --- | --- | --- | --- |
|  |  |  | **28s** | **COI** |
| 12439 | *Chartocerus* sp. 1 | 21 | KP771935 | - |
| 12440 | *Chartocerus* sp. 2 |  | KP771936 | - |
| 12441 | *Dendrocerus* sp. | 20 | KP771937 | - |
| 12442 | *Prochiloneurus* sp. |  | KP771938 | - |
| 12443 | *Pachyneuron* sp. | 3 | KP771939 | KP771959 |
| 12444 | *Lamennaisia* sp. | 2 | KP771940 | - |
| 12445 |  |  | KP771940 | - |
| 12446 |  |  | KP771940 | - |
| 12447 |  |  | KP771940 | - |
| 12448 |  |  | KP771940 | - |
| 12449 | *Anagyrus aurantifrons* | 1 | KP771941 | KP771960 |
| 12450 |  |  | KP771941 | KP771961 |
| 12451 | *Anagyrus aurantifrons* | 6 | KP771941 | KP771960 |
| 12452 |  |  | KP771941 | KP771961 |
| 12453 |  |  | KP771941 | - |
| 12454 |  |  | - | KP771960 |
| 12455 | *Aenasius comperei* | 4 | KP771944 | KP771964 |
| 12456 |  |  | KP771945 | - |
| 12457 |  |  | KP771944 | KP771964 |
| 12458 | *Anagyrus aurantifrons* | 5 | KP771941 | KP771961 |
| 12459 | *Lamennaisia* sp. |  | KP771940 | - |
| 12460 | Proctotrupoidea |  | KP771946 | - |
| 12461 | *Anagyrus aurantifrons* |  | KP771941 | KP771960 |
| 12462 | Cynipoidea |  | KP771947 | - |
| 12463 | Proctotrupoidea |  | KP771946 | - |
| 12464 | *Coccophagus* sp. | 17 | KP771948 | - |
| 12465 | *Anagyrus* sp. 1 | 19 | KP771942 | KP771962 |
| 12466 |  |  | KP771942 | KP771962 |
| 12469 | *Chartocerus sp. 1* | 18 | KP771935 | - |
| 12472 |  |  | KP771935 | - |
| 12473 |  |  | KP771935 | - |
| 12474 | *Rhopus notuis* |  | KP771949 | KP771965 |
| 12475 |  |  | KP771949 | KP771966 |
| 12476 |  |  | KP771949 | KP771965 |
| 12477 |  |  | KP771949 | KP771965 |
| 12478 | *Chartocerus* sp. 1 |  | KP771935 | - |
| 12480 | *Anagyrus aurantifrons* | 6 | KP771941 | KP771960 |
| 12481 | *Anagyrus* sp. 1 | 18 | KP771942 | - |
| 12482 | *Anagyrus* sp. 2 |  | KP771943 | KP771963 |
| 15065 | *Anagyrus* sp. 1 | 30 | KP771942 | - |
| 15066 |  |  | KP771942 | - |
| 15067 |  |  | KP771942 | - |
